# The Physician and the Homeopath: Their Relation in Practice

**Published:** 1903-09

**Authors:** 


### TEXAS MEDICAL JOURNAL.

AUSTIN, TEXAS.

A MONTHLY JOURNAL OF MEDICINE AND SURGERY.

EDITED AND PUBLISHED BY
F. E. DANIEL, M. D.

ASSOCIATE EDITORS:

WITTEN BOOTH RUSS, M. D., San Antonio, Texas.

P. M. PAYNE, M. D., Brownwood, Texas.

Published Monthly at Austin, Texas. Subscription price $1.00 a year in advance.

Eastern Representative: John Guy Monihan, St. Paul Building, 220 Broadway, New York City.

Official organ of the the West Texas Medical Association, the Houston District Medical Association, the Austin District Medical Society, the Brazos Valley Medical Association, the Galveston County Medical Society, and several others.

### THE PHYSICIAN AND THE HOMEOPATH: THEIR RELATION IN PRACTICE.

**THE PRINCIPLES OF ETHICS INVOLVED.**

The time-honored "Code of Ethics" of the medical profession of America was revised at the New Orleans meeting of the American Medical Association, May, 1903; or rather it was abolished, and what is called "Principles of Medical Ethics" was adopted instead. This has been published by the *Journal of the American Medical Association,* and issued by the American Medical Association in pamphlet form for distribution to State and county medical societies for the information and guidance of members. The following extract from the preface or introduction to the Principles I reproduce from the pamphlet for the information of the readers of the "Red-Back," and as a sort of text, from which I proceed to preach a small preachment on "The Physician in His Relation to the Homeopath in Practice":

"The caption 'Principles of Medical Ethics' has been substituted for 'Code of Medical Ethics.' Inasmuch as the American Medical Association may be conceived to hold a relation to the constituent State associations analogous to that of the United States through its constitution to the several States, the committee deemed it wiser to formulate the principles of medical ethics without definite reference to code or penalties. Large discretionary powers are thus left to the respective State and Territorial societies to form such codes and establish such rules for the professional conduct of their members as they may consider proper, provided, of course, that there shall be no infringement of the established ethical principles of this Association."

It will be seen that "large discretionary powers are left to State and Territorial societies," etc. (see last clause above) ; but no discretion is left to county or district societies; hence it devolves upon our State Medical Association to expound the law for the local societies, and at next meeting it must be attended to. A copy of the pamphlet may be had free of charge by applying to Dr. G. H. Simmons, Secretary American Medical Association and editor of its journal, Chicago, or to any of the Councilors of the Texas State Medical Association. It will be seen that the "Principles" says nothing about consultation with homeopaths, nor that "their sectarian title makes them quacks," as did the old code, but, instead, it says:

"CHAPTER II.

"THE DUTIES OF PHYSICIANS TO EACH OTHER AND TO THE PROFESSION AT LARGE.

"ARTICLE I.—DUTIES FOR THE SUPPORT OF PROFESSIONAL CHARACTER.

*"Obligation to Maintain Honor of the Profession.*

"SECTION 1. Everyone on entering the profession, and thereby becoming entitled to full professional fellowship, incurs an obligation to uphold its dignity and honor, to exalt its standing and to extend the bounds of its usefulness. It is inconsistent with the principles of medical science and it is incompatible with honorable standing in the profession for physicians to designate their practice as based on an exclusive dogma or a sectarian system of medicine."

It is thus left to the physician to decide in his own mind whether or not he can consult with one whose sectarian system of medicine is incompatible with honorable professional standing; but the question is: What constitutes a consultation? In the sense in which "consultation" is generally understood, it would be rank nonsense, utterly useless for two practicers (not "practitioners;" I hate that word—it is senseless. A "practitioner" is, or ought to be, one who

"practitions," whatever that may mean. A "practicer" is one who "practices"), who argue from opposite premises, to expect to meet on common ground. One or the other must abandon altogether his precepts and doctrines. There can be no "consultation." One or the other must yield, and adopt the principles of the other. But cases arise where a physician is asked to meet a homeopath in the sick room; and the question arises: What is the principle of ethics that shall govern the physician in a case like this: For instance, two homeopaths are up against a breech presentation, and confess their inability to deliver. The family send for two physicians. One gives chloroform and the other delivers, and the family elect to retain the homeopaths in charge. The physicians are dismissed. I do not think that the physicians, having answered the call, violated any principle of ethics in delivering the case; it was an "emergency," and would seem to be fully covered by the following clause from the "Principles":

"ARTICLE III.—THE DUTIES OF PHYSICIANS IN REGARD TO CONSULTATIONS.

*"The Broadest Humanity in Emergencies Required.*

"SECTION 1. The broadest dictates of humanity should be obeyed by physicians whenever and wherever their services are needed to meet the emergencies of disease or accident."

As usual, the physician is called on to make all the sacrifice, to take all the risk. Why should he "obey the broadest dictates of humanity," come in contact with those who, the Principles of Ethics of his profession say, are not in honorable professional standing by reason of their exclusive dogma, when the family do not obey the dictates of justice, fairness and honorable dealing; but who cling to the homo who has not served them, who confesses his inability to do what he undertook to do, what he was expected to do, what he was sent for to do, and dismiss the two physicians who have made so much sacrifice, at "the dictates of humanity," and saved two precious lives? It is an outrage; and yet public opinion and the "Principles of Ethics" of the profession demand that the physician shall be again and again subjected to it.

When the law does not cover any particular case the case should, and generally does, become "a law unto itself." The principle of ethics that should apply here is: The physician when sent for should demand that the homos, confessing their inability to handle the case, should be sent away before he consents to touch it, and, having delivered the child, he should, in common justice, as well as for the safety of mother and child and the protection of his own reputation, be retained in charge till attendance is no longer needed. He should receive the full fee, and the failures nothing. I don't hesitate to say that that is the "principle" that always governed me when in practice, and would govern me now, were I in practice. It is asking too much of a physician to expect him to submit to such use and abuse.

I do not say, however, that the physicians did not violate the principles of ethics in going to the case under the circumstances. A case in point recently occurred in Austin. It was the subject of discussion at the late meeting of the Travis County Medical Society, referred to elsewhere in this issue. Meantime there is a bylaw on the books of the Texas State Medical Association reciting, in effect, and without equivocation, that "if any member shall consult with a homeopath he shall be expelled." It is "up to" them to define "consultation," and to rule whether or not a member shall be subjected to such shameful treatment as in the above case because "humanity" demands it. I say no! A physician has rights, and he should defend them. If a homeopath can not handle a breech presentation he should get out, or be sent out, and a physician should be employed who can do so, and when he does he should be retained and paid. Unless the homo is dismissed, he should politely, but firmly, decline to be made use of in any such unreasonable and unjust manner. In brief, *my* construction of the principles of ethics is: A physician can have no relations whatever with a homeopath *as a physician,* under any circumstances, as long as he calls himself a homeopath, advertises himself as such, and gets practice by virtue of such representations. Should reputable homeopaths drop the absurd title, and call themselves "physicians" only, no one would care what "methods" they practice, nor would any questions be asked. Many of them would be eligible to membership in the State Medical Association, and would be admitted if they desired it.

